# Supernumerary Tooth Patterns in Non-Syndromic White European Subjects

**DOI:** 10.3390/dj11100230

**Published:** 2023-09-25

**Authors:** Eva Henninger, Luca Friedli, Miltiadis A. Makrygiannakis, Vasileios F. Zymperdikas, Moschos A. Papadopoulos, Georgios Kanavakis, Nikolaos Gkantidis

**Affiliations:** 1 Department of Orthodontics and Dentofacial Orthopedics, School of Dental Medicine, University of Bern, CH-3010 Bern, Switzerland; eva.henninger@unibe.ch (E.H.); luca.friedli@unibe.ch (L.F.); 2 Department of Orthodontics, School of Dentistry, National and Kapodistrian University of Athens, GR-11527 Athens, Greece; mimakr@dent.uoa.gr; 3 Department of Orthodontics, Faculty of Dentistry, School of Health Sciences, Aristotle University of Thessaloniki, GR-54124 Thessaloniki, Greece; vzymperdikas@dent.auth.gr (V.F.Z.); mikepap@dent.auth.gr (M.A.P.); 4 Department of Orthodontics and Pediatric Dentistry, UZB—University School of Dental Medicine, University of Basel, CH-4056 Basel, Switzerland; georgios.kanavakis@unibas.ch

**Keywords:** supernumerary tooth, odontogenesis, white people, non-syndromic, permanent dentition

## Abstract

Supernumerary teeth form at an incidence of about 3% in the population, with differences among races and various clinical consequences. Information on detailed patterns, and especially on white subjects, is scarce in the literature. Therefore, we aimed to investigate the patterns of non-syndromic permanent supernumerary teeth in a white European population. A record review was performed in different orthodontic clinics and identified 207 eligible individuals with 258 supernumerary teeth. Approximately 80% of the subjects had one supernumerary tooth, while 15% had two. Supernumerary tooth formation was more often evident in males (male/female: 1.65). However, there was no sexual dimorphism in its severity. The following pattern sequences, with decreasing prevalence order, were observed in the maxilla: 21 > 11 > 12 > 18 > 28 and in the mandible: 34 > 44 > 35 > 45 > 42. Supernumerary teeth were most often unilaterally present, without sexual dimorphism. In the maxilla, they were more often anteriorly present, whereas in the mandible, an opposite tendency was observed. Supernumerary teeth were consistently more often observed in the maxilla than in the mandible; 74% were impacted, 80% had normal orientation (13% horizontal, 7% inverted), and 53% had normal size. The present thorough supernumerary tooth pattern assessment enables a better understanding of this condition with clinical, developmental, and evolutionary implications.

## 1. Introduction

A supernumerary tooth is defined as a tooth or odontogenic structure formed additionally to the normal deciduous or permanent dentition that can erupt or remain unerupted [1,2,3]. Supernumerary teeth can occur unilaterally or bilaterally, whereas more than three supernumerary teeth are formed in only 4.6% of cases. Single supernumerary teeth are mostly present in the maxillary incisor region (sometimes referred as mesiodens), whereas multiple supernumerary teeth most often occur in the premolar region [4,5]. Most supernumerary teeth (75%) are conical and usually develop roots. They may erupt and be in an inverted position [6].

The prevalence of non-syndromic supernumerary teeth in humans varies between 1.2 and 6.0% for the permanent dentition and between 0.3 and 0.8% for the deciduous dentition [5,7,8]. In the permanent dentition, males have a 1.37 times higher risk of having supernumerary teeth than females [9]. The prevalence also differs depending on racial background, with higher occurrence reported in Asian than in white populations [10,11].

Several syndromes like Down’s syndrome, cleidocranial dysplasia, familial adenomatous polyposis, or Gardener’s syndrome have been associated with supernumerary teeth. However, supernumerary teeth are often present as isolated findings, without any related anomalies [1,12,13,14].

Various theories have been reported concerning the aetiology of supernumerary teeth [9]. The most common theory attributes the occurrence of additional tooth structures to dichotomy of a tooth bud or to a hyperactive dental lamina, with various environmental, epigenetic, and genetic factors also considered to be involved [1,5,15,16,17]. At a molecular and genetic level, different studies have reported genes and signalling pathways associated with tooth morphogenesis such as Wnt, FGF, BMP, and Shh. Nevertheless, the molecular mechanisms of supernumerary tooth formation are still unclear [17,18,19,20].

Most supernumerary teeth are unidentified or misdiagnosed unless they develop complications like ectopic tooth eruption, impaction or delayed eruption of the adjacent teeth, crowding of teeth, odontogenic cyst formation, paraesthesia, displacements, midline diastemas, delayed exfoliation of primary teeth, or aesthetic impairments [15,21,22]. Due to the associated clinical complications and the subsequent impact on oral-health-related quality of life, early detection, multidisciplinary treatment approaches, and long-term follow-ups are required in certain cases [23,24,25]. The treatment need and type depend on several factors, such as the age, the type and position of the supernumerary tooth, and the clinical characteristics of the case [26].

Knowledge about the exact patterns of supernumerary teeth can facilitate the proper diagnosis and prevention of related dental malformations and provide a better understanding of this condition. To our knowledge, few existing studies explore the supernumerary tooth patterns using a considerable patient sample. We identified one study on a Turkish population [27], two studies on Chinese subjects [28,29], and one study performed in Spain [4], without information on the racial background of the tested sample. There are very few studies assessing well-defined and documented white European populations, and these are usually of limited sample size [30]. Moreover, we did not find any studies that investigated the exact patterns of supernumerary tooth formation in a sample per individual.

Therefore, the aim of the present study was to investigate the patterns of permanent supernumerary tooth formation in a well-documented, large orthodontic sample of a white European population.

## 2. Materials and Methods

### 2.1. Ethical Approval

Ethical approval was obtained from the Research Ethics Committees of the cantons of Bern, Neuchatel, Basel, and Jura, Switzerland (protocol nr: 2022-00399, date of approval: 20 June 2022) and the Institutional Ethics and Research Committees of the Dental Schools of the National and Kapodistrian University of Athens (protocol nr: 518/05.09.2022, date of approval: 13 October 2022) and the Aristotle University of Thessaloniki, Greece (protocol nr: 182/10.02.2023, date of approval: 16 March 2023). The methods were carried out in accordance with the relevant guidelines and regulations. All participants signed an informed consent form prior to the use of their data in the study.

### 2.2. Study Sample

In the context of a retrospective study design, the patient archives of the Department of Orthodontics at the University of Bern, the University of Basel, two private practices in Switzerland, the National and Kapodistrian University of Athens, and the Aristotle University of Thessaloniki were analysed to identify individuals with supernumerary teeth. More than 10,000 archived patient files were searched for different time spans between 2002 and 2023, depending on location. Researchers in the locations of sample collection searched the archives consecutively (medical and dental history, intraoral and extraoral photos, radiographs), identified the individuals who fulfilled the eligibility criteria, and anonymized the data for further assessment. For each individual, the existing pre-treatment panoramic and cephalometric radiographs and any additional diagnostic radiographs (e.g., periapical or cone beam computed tomography) were retrieved. The pre-treatment dental models, intraoral or extraoral photos, and the medical and dental patients’ history were also examined to enhance the diagnostic ability, if needed. The pre-treatment panoramic and cephalometric radiographs, dental models, and intraoral and extraoral photos comprise standard records available for all patients who undergo orthodontic treatment.

In total, 207 individuals compatible with the inclusion criteria were found. The sample size was based on availability and empirical evidence, and it is considered satisfactory for the study purpose [30].

The tooth sequence of the included subjects was identified in the coded panoramic radiographs and registered in an Excel spreadsheet (Microsoft Excel, Microsoft Corporation, Redmond, WA, USA) together with the associated patient data, namely age and sex.

The following inclusion criteria were applied:

- Individuals older than 8 years of age and younger than 50 years of age when the pre-treatment radiograph was obtained. In cases younger than 12 years old when the pre-treatment radiograph was obtained, any radiographs obtained in older ages were examined to confirm potential late-forming supernumerary teeth (no such case was detected).
- European ancestry (white subjects). This was the major racial type represented in the searched archives. Other racial backgrounds were quite variable and largely underrepresented to form reasonable groups.
- Individuals with supernumerary teeth.
- No syndromes, systemic diseases, or any other defects that affect craniofacial morphology as reported in the subjects’ medical records.
- No extensive dental restorations that may affect craniofacial morphology.
- High-quality panoramic radiographs or cone beam computed tomography for identification of supernumerary teeth.
- No intervention that could influence craniofacial morphology, such as orthodontic treatment, prior to image acquisition.
- No other severe dental anomaly in tooth size or form in any tooth apart from third molars.

### 2.3. Data Collection

**Figure 1:**
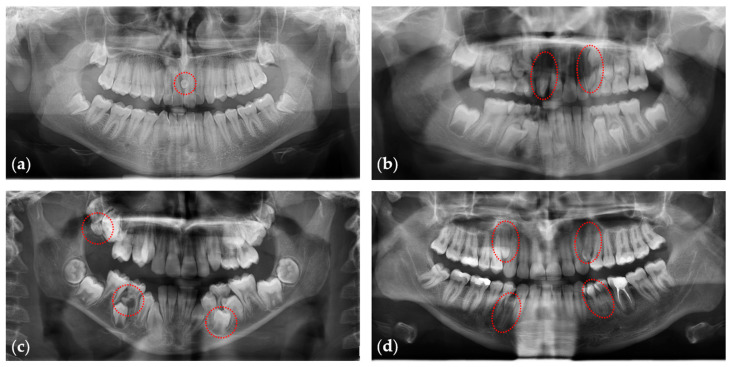
Panoramic radiographs showing individuals with (**a**) one, (**b**) two, (**c**) three, or (**d**) four supernumerary teeth, indicated by dashed red circles.

Further evaluation of the anonymized radiographs was performed by two researchers (E.H, L.F.) to verify the data extraction procedure. All panoramic radiographs were viewed on a screen to identify tooth patterns. For this purpose, hard copy (analogical) radiographs were scanned using an Epson Perfection V700 scanner with a resolution of 600 dpi at a scale of 1:1 and saved in .tiff (tagged image file format) format (Figure 1). Both researchers re-assessed the data extraction procedure of the entire sample one month after the first assessment, and any disagreements were resolved through consensus and consultation with the last author. All data were recorded in an Excel spreadsheet.

A numerical coding system analogous to the TAC system [31] that we used previously for tooth agenesis [32] was implemented to investigate the patterns of supernumerary teeth. Through this, a binary value was assigned to each tooth, providing a unique numeric value for each supernumerary tooth pattern.

The eruption status of supernumerary teeth (erupted or impacted), their orientation (normal, horizontal, or inverted), and size (normal, small, or not assessable) were visually assessed on the available radiographs by two researchers (E.H. and L.F.) independently. Any disagreements were resolved through consensus and consultation with the last author. Regarding orientation, a tooth was considered horizontal or inverted if it deviated more than 45% from the normal or horizontal position, respectively. A tooth was classified as small if it was judged to be at least 30% smaller than the corresponding normal tooth size. If the available information did not permit a reliable assessment, the tooth was categorized as not assessable.

### 2.4. Statistical Analysis

Descriptive and comparative statistics were applied on the study data. Statistical analysis was carried out by using the IBM SPSS for Windows (Version 28.0. Armonk, NY, USA: IBM Corp). Chi-square tests were performed through the following web-based calculator: Chi-Square Test Calculator, Social Science Statistics, https://www.socscistatistics.com/tests/chisquare2/default2.aspx (accessed on 22 June 2023). The level of significance for the study was set at 0.05.

## 3. Results

In total, 207 patients with 258 supernumerary teeth were identified in the searched archives after applying the eligibility criteria. In the sample, there were two males with a supernumerary 11 (mesiodens) who also showed agenesis of one lower second premolar.

Approximately 80% of the subjects had one supernumerary tooth, followed by approximately 15% who had two supernumerary teeth. There was no sexual dimorphism in the severity of supernumerary tooth formation (*p* = 0.105), although only a few males with more than two supernumerary teeth were detected in the sample (Table 1). Supernumerary tooth formation was more often evident in males, with a male to female ratio of 1.65 (chi-square test: *p* = 0.011) (Table 1). There were 78 females (age: 13.1 ± 7.8 years) and 129 males (age: 12.1 ± 4.5 years) with 90 and 168 supernumerary teeth, respectively. There was no difference in the age of the sexual groups (unpaired t-test, *p* = 0.241). Regarding the severity of the condition, 1.25 ± 0.58 supernumerary teeth were found on average per individual (males: 1.30 ± 0.67, females: 1.15 ± 0.36 supernumerary teeth; Mann–Whitney U test, *p* = 0.209) (Table 1).

The number of supernumerary teeth per tooth type and the prevalence according to the total number of supernumerary teeth in the entire sample, as well as in male and female groups, is provided in Table 2. In the total sample, the following sequences, with decreasing order, were observed in the maxilla: 21 > 11 > 12 > 18 > 28 and in the mandible: 34 > 44 > 35 > 45 > 42. Similar outcomes were observed for each sex separately (males, maxilla: 21 > 11 > 12 > 18 > 28 and mandible: 34 > 44 > 35 > 45 or 31; females, maxilla: 21 or 22 > 11 or 12 > 18 and mandible: 34 > 35 or 44 or 45 > 42 or 48).

Considering all supernumerary teeth formed per individual, as well as their location, the most common patterns of supernumerary teeth overall and the five most common patterns in males and in females are presented in Table 3. In all groups, supernumerary single upper incisors are consistently present in the first four most common patterns.

Overall, as well as when considering the most common patterns (Table 3) and sexual groups separately, supernumerary teeth were present more often unilaterally than bilaterally (Table 4, *p* < 0.05 in all but one cases). Overall, unilateral occurrence was present 5.9 times more often in the maxilla and 3.5 times more often in the mandible (*p* = 0.19, Table 4). There was no sexual dimorphism in terms of bilateral versus unilateral occurrence for both jaws (maxilla: *p* = 0.331, mandible *p* = 0.425).

Regarding the anteroposterior location of the supernumerary teeth, in the maxilla, they were more often present anteriorly (*p* < 0.05), whereas in the mandible, an opposite tendency was observed (*p* > 0.05, Table 5). In the maxilla, males presented anterior location 7.0 times more often, whereas for females, this was only 2.2 times (*p* = 0.006). No sexual dimorphism was present in the mandible for anteroposterior location (*p* = 0.556). Supernumerary teeth were more often observed in the maxilla than in the mandible in all groups (*p* < 0.001, Table 6), with no differences between sexes (*p* = 0.170).

Concerning the supernumerary tooth characteristics, 74% were impacted (*p* < 0.001), 80% had a normal orientation (13% horizontal, 7% inverted; *p* < 0.001), and 53% had normal size (*p* = 0.129). Similar outcomes were evident for each sex separately (Table 7).

## 4. Discussion

The present study explored the patterns of permanent supernumerary teeth in a well-documented white European orthodontic population, consisting of 207 individuals with 258 supernumerary teeth in total. Most individuals had one supernumerary tooth, followed by two, with higher predisposition for males. Supernumerary single upper incisors were consistently present in the first four most common patterns of all groups. Supernumerary teeth appeared mostly unilaterally, and they were more often detected in the maxilla than in the mandible. In the maxilla, they were more often present anteriorly, in contrast to the mandible. Most supernumerary teeth were impacted and had normal orientation, and almost half of them had normal size.

To our knowledge, the available evidence on the patterns of supernumerary teeth is scarce, since several previous studies—some with considerable samples—reported their data using the tooth as the unit of analysis and not the individual [4,27,28,29,30]. Therefore, the patterns of individuals with more than one supernumerary tooth were not considered, and additionally, the single tooth pattern outcomes were biased because of this. Moreover, we were not able to identify any previous study investigating a considerable sample from a white racial background [4,27,28,29,30]. The available studies either did not report on the racial background of the included individuals [4] or had limited sample size [4,27,28,29,30]. Therefore, the present sample of 207 well-documented white patients seems to be unique in the literature and allowed a thorough investigation of supernumerary tooth patterns. The archived records were comprehensive and included detailed medical and dental histories, as well as facial and intraoral photographs and radiographs. Additionally, patients who underwent orthodontic treatment were monitored for a certain period, and the orthodontic specialists responsible for data collection were trained to identify abnormalities in craniofacial development. Therefore, there is a minimal likelihood of individuals with syndromes or other pathologies that impact craniofacial morphology being included in the sample.

Since most supernumerary teeth remain undiagnosed unless they develop complications [24,25], from a clinical point of view, the knowledge of the patterns of supernumerary teeth enables us to foresee possible occurrences and is therefore crucial for proper diagnosis and timely treatment planning. Moreover, it assists researchers in planning future clinical studies on the topic. From an anthropological point of view, these studies offer a better understanding of the development of the dentition with potential biological and evolutionary implications [32,33,34,35].

In the maxilla, supernumerary teeth were more often anteriorly present, whereas in the mandible, an opposite tendency was found. The same findings were evident in various previous studies [9,16,18,27,28,29], except from one that found a higher number of supernumerary teeth in the distomolar region [4]. Per jaw, supernumerary teeth were more often observed in the maxilla than in the mandible in all groups, which is in line with previous studies [4,16,18,21,27,29].

Concerning the supernumerary tooth characteristics, 74% were impacted, 80% had a normal orientation, and 53% had normal size. Similar findings have been reported previously, with most studies focusing on the eruption status of the supernumerary teeth [4,16,18,21,27,29]. The facts that approximately half of the teeth did not have a normal size and that shape abnormalities were often evident underline the need for careful evaluation during treatment planning, through CBCT if needed, especially when the teeth are impacted and a decision for extraction needs to be taken [36,37]. Novel AI tools can assist dentists in the diagnosis and treatment planning of such challenging cases [36,38].

In the present sample, supernumerary tooth formation was more often evident in males, which is in agreement with previous studies [9]. Interestingly, studies investigating tooth agenesis patterns revealed an opposite trend of similar magnitude, showing higher agenesis prevalence in females compared to males [39]. Despite the sexual predisposition in the incidence of these conditions, we did not detect sexual dimorphism in the severity of supernumerary tooth formation, and the same was evident for tooth agenesis [33]. When comparing the patterns of supernumerary teeth between males and females, males show anterior supernumerary teeth more often compared to females. This is consistent with other studies testing samples of different racial backgrounds [27,28,29,33]. The aforementioned findings strengthen the hypothesis of genetic involvement in supernumerary tooth formation and suggest an opposite sexual effect on the incidence of agenesis versus supernumerary tooth formation, with no impact on the severity of these conditions.

Regarding the severity of tooth number abnormalities, fewer than 1 out of 1000 individuals show three of more supernumerary teeth, with an overall prevalence of about 3% [5]. Compared to agenesis, which has a prevalence of 6.4%, with 4.6 out of 1000 individuals showing three or more missing teeth [36], we can conclude that multiple supernumerary teeth comprise a rarer finding than multiple agenesis. Moreover, supernumerary teeth were more often present unilaterally than bilaterally, without any sexual dimorphism [27]. This is in contrast to individuals with agenesis, where an opposite trend was observed [32]. Individuals with third molar agenesis also showed more frequent bilateral occurrence [33,40]. These observations indicate more generalized and impactful effects of the genetic or other factors leading to tooth agenesis as compared to supernumerary tooth formation. This might be related to the biological mechanism of tooth number reduction, observed during human evolution, which might still be active and continue to regulate the number of teeth and facial morphology in a coordinated manner [34,35]. Recent evidence suggested that in modern humans, the number of teeth that are formed in a dentition is associated with facial size and shape, and this also concerns third molar formation [32,33,35,41]. At present, no such information is available regarding supernumerary teeth, but previous studies indicated strong genetic involvement in tooth development from different perspectives [42].

The present study has several strengths, which have been reported above. One limitation is the inclusion of only orthodontic patients, who may vary in the severity of supernumerary tooth incidence compared to the general population. However, several of our findings are in line with those of previous studies on different populations, adding validity to the outcomes. This, along with the endemic occurrence of malocclusion and the widespread offer of orthodontic services in modern societies [43], might justify the extrapolation of the present findings to the general population. The age range that we considered was limited from 8 to 50 years old for availability reasons. In the youngest individuals of the sample, as supernumerary teeth may have developed at a later age, it is possible to underestimate supernumerary tooth development. To overcome this limitation, any available records of these individuals from later stages were re-examined to confirm diagnosis. Another limitation might be that no detailed records or demographic data of the excluded individuals were kept. This was decided because the aim of the study was to thoroughly investigate supernumerary tooth patterns and not the prevalence of the condition in the population. However, the reported male/female ratio might have been slightly underestimated due to this, since usually, slightly more females seek orthodontic treatment [43]. Finally, supernumerary tooth formation can vary in severity and pattern depending on geographic area or ancestry [10,11,27,29,30]. To minimize confounding, only white European subjects were included in this study, since they were overrepresented in the searched archives. Therefore, the present findings might not be generalizable to other types of ancestry.

## 5. Conclusions

The present study explored the patterns of permanent supernumerary teeth in a well-documented white European population. Most individuals had one supernumerary tooth, followed by two, with higher predisposition for males, but no sexual dimorphism in the severity of the condition was observed.

Although genetic involvement was denoted by the data, when compared to findings on tooth agenesis, the present observations suggested more localized and less impactful effects of the genetic or other factors involved in supernumerary tooth formation. From a clinical point of view, the knowledge of the patterns of supernumerary teeth enables us to foresee possible occurrences and is, therefore, crucial for proper diagnosis and timely treatment planning.

## Figures and Tables

**Table 1:** Number of supernumerary teeth per individual in males and females.

Nr. of Supernumerary Teeth	Males	Females	*p*-Value *
1	101 (78.3%)	66 (84.6%)	*p* = 0.105
2	21 (16.3%)	12 (15.4%)	
3	3 (2.3%)	0	
4	4 (3.1%)	0	

* Chi-square test between males and females.

**Table 2:** Number of supernumerary teeth per tooth type and prevalence (%) according to the total number of supernumerary teeth in the entire sample, as well as in male and female groups separately.

Maxilla																
Teeth	18	17	16	15	14	13	12	11	21	22	23	24	25	26	27	28
Males (*n* = 119)	5 3.0%	-	1 0.6%	-	2 1.2%	1 0.6%	20 11.9%	28 16.7%	37 22.0%	11 6.5%	3 1.8%	3 1.8%	2 1.2%	1 0.6%	1 0.6%	4 2.4%
Females (*n* = 63)	10 11.1%	1 1.1%	1 1.1%	-	2 2.2%	-	9 10.0%	9 10.0%	11 12.2%	11 12.2%	1 1.1%	1 1.1%	1 1.1%	-	-	6 6.6%
Total (*n* = 182)	15 5.8%	1 0.3%	2 0.7%	-	4 1.5%	1 0.3%	29 11.2%	37 14.3%	48 18.6%	22 8.5%	4 1.5%	4 1.5%	3 1.1%	1 0.4%	1 0.4%	10 3.8%
**Mandible**																
**Teeth**	**48**	**47**	**46**	**45**	**44**	**43**	**42**	**41**	**31**	**32**	**33**	**34**	**35**	**36**	**37**	**38**
Males (*n* = 49)	2 1.2%	-	-	4 2.4%	9 5.3%	1 0.6%	3 1.8%	1 0.6%	4 2.4%	2 1.2%	3 1.8%	11 6.5%	6 3.6%	-	-	3 1.8%
Females (*n* = 27)	2 2.2%	-	-	4 4.4%	4 4.4%	1 1.1%	2 2.2%	1 1.1%	1 1.1%	2 2.2%	-	5 5.6%	4 4.4%	1 1.1%	-	-
Total (*n* = 76)	4 1.6%	-	-	8 3.1%	13 5.0%	2 0.8%	5 1.9%	2 0.8%	5 1.9%	4 1.6%	3 1.2%	16 6.2%	10 3.9%	1 0.4%	-	3 1.2%

**Table 3:** The most common patterns of supernumerary teeth overall and the five most common patterns in males and in females.

Group	Most Common Patterns	Frequency (%)	Supernumerary Teeth	Bilateral	Unilateral
Total sample	1	35/207 (16.90%)	21		*
	2	26/207 (12.56%)	11		*
	3	24/207 (11.59%)	12		*
	4	19/207 (9.17%)	22		*
	5	10/207 (4.83%)	11,21	*	
	6	9/207 (4.34%)	18		*
	7–8	6/207 (2.89%)	34 or 35		**
	9–12	5/207 (2.41%)	18, 28 or 31 or 42 or 44, 34	**	**
Males	1	26/129 (20.15%)	21		*
	2	19/129 (14.72%)	11		*
	3	16/129 (12.40%)	12		*
	4	9/129 (6.97%)	22		*
	5	8/129 (6.20%)	11,21	*	
Females	1	10/78 (12.82%)	22		*
	2	9/78 (11.53%)	21		*
	3	8/78 (10.25%)	12		*
	4	7/78 (8.97%)	11		*
	5	6/78 (7.69%)	18		*

* Each asterisk indicates a single pattern categorization.

**Table 4:** Symmetry in supernumerary tooth formation in terms of laterality for each jaw separately (not considering the 6 individuals who had supernumerary teeth in both jaws).

Group	Maxilla			Mandible		
	Bilateral	Unilateral	*p*-Value *	Bilateral	Unilateral	*p*-Value *
Total sample	22	130	*p* < 0.001	11	38	*p* < 0.004
Males	14	83	*p* < 0.001	7	19	*p* < 0.089
Females	8	47	*p* < 0.001	4	19	*p* < 0.018

* Chi-square test.

**Table 5:** Symmetry in supernumerary tooth formation in terms of anteroposterior location for each jaw separately (not considering the 6 individuals who had supernumerary teeth in both jaws).

Group	Maxilla				Mandible			
	Anterior (13–23)	Posterior (14/24–18/28)	*p*-Value *	Anterior and Posterior	Anterior (33–43)	Posterior (34/44–38/48)	*p*-Value *	Anterior and Posterior
Total sample	122	29	*p* < 0.001	1	17	32	*p* < 0.123	0
Males	84	12	*p* < 0.001	1	10	16	*p* < 0.402	0
Females	38	17	*p* < 0.041	0	7	16	*p* < 0.172	0

* Chi-square test.

**Table 6:** Location of supernumerary tooth formation according to jaw.

Group	Maxilla	Mandible	Both	*p*-Value *
Total sample	152 (73.42%)	49 (23.67%)	6 (2.89%)	*p* < 0.001
Males	97 (75.19%)	26 (20.15%)	6 (4.65%)	*p* < 0.001
Females	55 (70.51%)	23 (29.48%)	0 (0.00%)	*p* < 0.001

* Chi-square test.

**Table 7:** Eruption status, orientation, and size of the supernumerary teeth, overall, in males, and in females. The numbers represent counts of supernumerary teeth.

Group	Eruption Status			Tooth Orientation				Tooth Size			
	Erupted	Impacted	*p*-Value *	Normal	Horizontal	Inverted	*p*-Value *	Normal	Small	*p*-Value *	NA
Total sample	67 (25.96%)	191 (74.03%)	*p* < 0.001	208 (80.62%)	33 (12.79%)	17 (6.58%)	*p* < 0.001	138 (53.48%)	102 (39.53%)	*p* = 0.129	18 (6.97%)
Males	47 (27.97%)	121 (72.02%)	*p* < 0.001	137 (81.54%)	18 (10.71%)	13 (7.73%)	*p* < 0.001	92 (54.76%)	62 (36.90%)	*p* = 0.107	14 (8.33%)
Females	20 (22.22%)	70 (77.77%)	*p* < 0.001	71 (78.88%)	15 (16.66%)	4 (4.44%)	*p* < 0.001	46 (51.11%)	40 (44.44%)	*p* = 0.647	4 (4.44%)

* Chi-square test, NA: not assessable.

## Data Availability

All data reported in the manuscript are provided in the submitted tables, figures, and in the main text. The datasets generated and/or analysed in the current study are available from the corresponding author on reasonable request.
